# Particulate Matter Mortality Rates and Their Modification by Spatial Synoptic Classification

**DOI:** 10.3390/ijerph16111904

**Published:** 2019-05-29

**Authors:** Jayeun Kim

**Affiliations:** Institute of Health and Environment, Seoul National University, Seoul 08826, Korea; ginakim@snu.ac.kr; Tel.: +82-2880-2756

**Keywords:** mortality, air pollution, synoptic weather, modifier

## Abstract

Air pollution levels are highly correlated with temperature or humidity, so we investigated the relationship between PM_10_ and the spatial synoptic classification (SSC) scheme on daily mortality, according to age group and season. Daily death data for 2000–2014 from Seoul, Korea, were acquired, and time-series analysis was applied with respect to season and to each of seven distinct SSC types: dry moderate (DM); dry polar (DP); dry tropical (DT); moist moderate (MM); moist polar (MP); moist tropical (MT); and transition (T). Modification effects were estimated for daily, non-accidental, cardiovascular, and respiratory mortality between PM_10_ and SSC types. The following SSC-type-specific increased mortalities were observed, by cause of death: non-accidental mortality: DT (1.86%) and MT (1.86%); cardiovascular mortality: DT (2.83%) and MM (3.00%); respiratory mortality: MT (3.78%). Based on simplified weather types, increased PM_10_ effects in non-accidental mortality rates were observed in dry (1.54%) and moist (2.32%) conditions among those aged 40–59 years and were detected regardless of conditions in other age groups: 60–74 (1.11%), 75–84 (1.55%), and 85+ (1.75%). The effects of particulate air pollution, by SSC, suggest the applicability of SSC to the comparison and understanding of acute effects of daily mortality based on weather type.

## 1. Introduction

Associations between particulate matter (PM) and mortality have been reported from several perspectives, such as cause of death [1,2,3,4,5,6], PM fraction size [1,7,8,9,10,11], study population variables including age [12,13,14], sex [15], or race and ethnicity [16], and varied study settings [4,7,9,17,18,19,20,21,22,23,24] including global estimates [25].

Looking into non-accidental, cardiovascular, and respiratory mortality further, several studies have refined the causes of death and investigated elevated PM levels with adverse associations with hypertensive mortality [1], stroke [2], myocardial infarction [3], intracerebral hemorrhage [4], breast cancer [5], and chronic obstructive pulmonary disease [6]. In addition, health effects related to different PM sizes have been investigated [1,9], and coarse particle distribution has been studied [7,8,9,10], as fraction size in the air is an important consideration for PM studies. To confirm the health effects of PM, cohort study design for long-term effects [17,19,20] and time series study designs for short-term effects [4,7,9,18,21,22,23,24,26] have been widely used.

In addition, the effect of temperature as a PM effect modifier has been studied [26,27,28], while other meteorological variables have received less attention. Although daily weather conditions are overwhelmingly dependent on temperature, the influence of relative humidity and other meteorological conditions should not be disregarded. Investigating health effects modified by PM by looking at all meteorological factors is likely to encounter redundancy, however, or become inefficient. Accordingly, we have hypothesized that the relationship between PM_10_ change and daily mortality may be influenced on a daily basis, depending on the degree of dryness or moisture on a given day.

Hence, to identify mortality sensitivities under varied daily weather conditions, and see if they included other variables [29] affecting daily mortality, we adapted the spatial synoptic classification (SSC) scheme to investigate the modification of PM_10_ effects, in terms of exposure to specific daily weather conditions. The SSC scheme is a relative and daily weather-type classification scheme, rather than an absolute classification system [30], and is now widely used in climatological and epidemiological investigations [31,32,33,34,35,36,37,38]. Previously, the relationship between the SSC and particulate air pollution had been considered in relation to lung cancer mortality [38] and atopic dermatitis [37], but the extent of mortality from PM pollution in relation to the applied SSC scheme has received limited attention. Therefore, in the study, we aimed to identify any modification effect on mortality rates between PM_10_ and SSC elements. We estimated the modified effects of PM_10_ by SSC type according to age group and season, adjusting for other meteorological conditions, air pollution, and time trends.

## 2. Materials and Methods

### 2.1. Mortality Data for Study Population

Death data for Seoul, South Korea, from 2000 to 2014, were used in this analysis. Death data are collated by Korea Statistics and include date of death, age, and sex; they also list the cause of death using the categories in the International Classification of Diseases, tenth Revision (ICD–10) (except for accidents, fatal injuries, and the deaths of foreign persons). Deaths from non-accidental mortality, that is, those classified using the ICD–10 standard as A00–R99, from cardiovascular mortality (I00–I99) and from respiratory mortality (J00–J99), were analyzed.

### 2.2. Air Pollution and Meteorological Exposure

Data regarding the daily concentrations of PM ≤10 μm in aerodynamic diameter (PM_10_), 8 h mean ozone concentrations (O_3_) (ppb, parts per billion), and 24 h mean nitrogen dioxide (NO_2_) concentrations (ppb) were collected from the Environmental Monitoring Center of the National Institute of Environmental Research, Korea. We obtained the daily SSC for Seoul, Korea, from the SSC archive (http://sheridan.geog.kent.edu/ssc.html). The SSC scheme allocates weather into one of six weather type categories: dry moderate (DM), dry polar (DP), dry tropical (DT), moist moderate (MM), moist polar (MP), and moist tropical (MT), with an additional transition type (T), used to indicate that, at the sampling moment, the weather was transitioning from one category to another.

Other meteorological data, including daily mean, maximum, and minimum temperatures, relative humidity, air pressure, and rainfall, were obtained from the Korean Meteorological Administration. Air pollution data were collected from 27 air pollution monitoring sites over the period 2000–2009 and from 25 air pollution monitoring sites over the period 2010–2013 by the National Institute of Environmental Research and, for 2014, collected from 39 air pollution monitoring sites by the Korea Environment Corporation (Air Korea). Hourly measurements for meteorological variables and for air pollution were collected, and daily mean, maximum and minimum exposure levels were calculated. The diurnal temperature range was derived as the difference between the daily maximum and daily minimum temperatures, and daily rainfall was expressed as a binary variable, in that days with precipitation >0 mm were identified as rainy days.

### 2.3. Statistical Methods

We performed a time-series study using Poisson generalized linear models, allowing for over-dispersion within the stratification of the seven distinct SSC types. If necessary, we combined several SSC types into one group; for example, (DM), (DP), and (DT) were combined into “dry” and (MM), (MP), and (MT) into “moist,” while (T) stood for “transition” from a temperature standpoint. Similarly, (DM) and (MM) were combined into “moderate,” (DP) and (MP) into “polar,” and (DT) and (MT) into “tropical,” while (T) stood for “transition,” from a humidity standpoint. In addition, to consider the cause of death in terms of age-specific effects, we separated the 40–59, 60–74, 75–84, and 85+ age groups out of the total mortality data. In order to evaluate seasonal influence, seasons were defined as spring (March–May), summer (June–August), fall (September–November), and winter (December–February).

Poisson generalized linear regression analyses allowing for over-dispersion were applied to the daily PM_10_ level, in relation to daily mortality, during the study period, and we investigated the modification effect of PM_10_ by SSC type, adjusting for meteorological variables including daily mean temperature, diurnal temperature range, relative humidity, air pressure, and rainfall. In addition, air pollution and time trends were adjusted: We applied moving average values for continuous variables for up to four consecutive days, including daily levels of PM_10_, considering the delayed effect. Long-term trends were controlled with natural cubic splines, with 7 degrees of freedom (*df*) per year, so that 105 (*df*) was obtained overall. In addition, daily mean temperature was controlled with natural cubic splines with 7 (*df*). When initially estimating PM_10_ health effects, the overall effect was estimated by adding four consecutive days of PM_10_ moving averages, as a linear explanatory variable in the Poisson regression model, for individual cause of death (Equation (1)). Afterwards, we estimated the modified effect of PM_10_ according to SSC types with an approach to quantity PM_10_ effect on mortality in different SSC strata (Equation (2)).

(a) Overall effect of PM_10_:
  (1) $$\begin{array}{c} \ln\left(E\left(y_{t}\right)\right)=\beta_{0}+\beta_{1}\left(PM_{10_{t:t-i}}\right)+\beta_{2}\left(temp_{t:t-i}\right)+\beta_{3}\left(DTR_{t:t-i}\right)+\beta_{4}\left(humid_{t:t-i}\right)\\ \;+\beta_{5}\left(press_{t:t-i}\right)+\beta_{6}\left(O_{3_{t:t-i}}\right)+\beta_{7}\left(NO_{2_{t:t-i}}\right)+factor\left(DOW_{t}\right)\\ \;+ns\left(time_{t},df\right). \end{array}$$
(b) Modified effect of PM_10_ according to SSC strata:
  (2) $$\begin{array}{c} \ln\left(E\left(y_{t}\right)\right)=\beta_{0}+\beta_{1}\left(PM_{10_{t:t-i}}\right):factor(SSC_{t,j})+\beta_{2}\left(temp_{t:t-i}\right)+\beta_{3}\left(DTR_{t:t-i}\right)+\beta_{4}\left(humid_{t:t-i}\right)\\ \;+\beta_{5}\left(press_{t:t-i}\right)+\beta_{6}\left(O_{3_{t:t-i}}\right)+\beta_{7}\left(NO_{2_{t:t-i}}\right)+factor\left(DOW_{t}\right)\\ \;+ns\left(time_{t},df\right). \end{array}$$

In Equations (1) and (2), *t* refers to the day of the observation; *i* refers to the lag; $E\left(y_{t}\right)$ denotes the estimated daily case numbers on day *t* among cause-specific mortality numbers for Seoul. Term (*df*) denotes the degrees of freedom; $PM_{10_{t:t-i}}$ is the moving average PM_10_ value between days *t* and *t − i*; $SSC_{t,j}$ is the factor value of *j* types of SSC on day *t*; $DOW_{t}$ is the day of the week on day *t*; and $time_{t}$ denotes the seasonality, using calendar days.

We calculated the relative risk percentage change, with its 95% confidence interval (CI), for daily, non-accidental, cardiovascular, and respiratory mortality associated with exposure to PM_10_ per interquartile range (IQR) increase, according to SSC type, age group, and season.

### 2.4. Ethics Approval

This study was approved by the Institutional Review Board of Seoul National University (IRB No. E1707/003-010) in Seoul, Republic of Korea.

## 3. Results

### 3.1. Descriptive Statistics of Mortality and Environmental Factors

Descriptive statistics for the daily mortality and environmental variables are summarized in Table 1. Overall, daily, cause-specific mortalities were as follows: non-accidental mortality (mean: 95.4 (SD: 12.3)), cardiovascular mortality (mean: 23.8 (SD: 5.7)), and respiratory mortality (mean: 6.7 (SD: 2.8)). The mortality rate was highest during winter for non-accidental (mean: 101.3 (SD: 12.5)), cardiovascular (mean: 25.8 (SD: 5.6)), and respiratory (mean: 7.3 (SD: 3.1)) mortality. Among the environmental covariates, mean temperature and relative humidity were highest during summer and lowest during winter, whereas the diurnal temperature range was higher during spring and fall than it was during either summer or winter. The daily PM_10_ level was high during spring and winter, whereas O_3_ level was high during summer. In terms of the days in each SSC category, DM and DP accounted for 2037 days (37.2%) and 1052 days (19.2%), respectively, during the fifteen-year study period (2000–2014). The highest proportions, by season, of the seven SSC types were as follows: spring, DM (604 days (43.8%)); summer, MT (490 days (35.5%)); fall, DM (737 days (54.0%)); and winter, DP (640 days (47.3%)).

**Figure 1:**
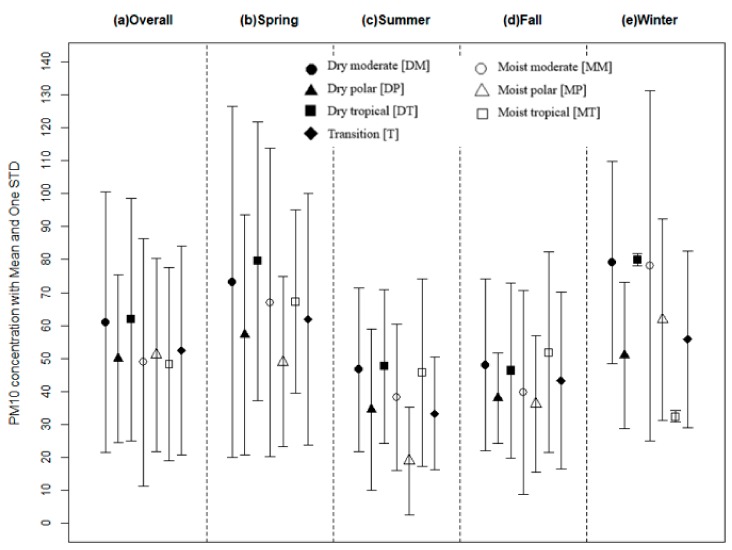
PM_10_ concentration distribution presented with the daily mean level and one standard deviation upon spatial synoptic classification and seasons between 2000 and 2014 in Seoul, South Korea.

Figure 1 shows the daily PM_10_ concentration distribution, with means and one standard deviation (SD), according to SSC type—including PM_10_ concentrations with seasonal breakdowns—between 2000 and 2014. In overall terms, mean PM_10_ value was higher in DM (mean: 61.0 (SD: 39.6)) and DT (mean: 61.8 (SD: 36.8)), while in seasonal terms, the PM_10_ level was higher for DM and DT during winter (mean: 79.5 (SD: 42.2)), and spring (mean: 73.2 (SD: 53.2)), respectively.

### 3.2. PM_10_ Daily Mortality Modification by Weather Type

Daily mortality due to the PM_10_ level was fitted in overall terms and according to SSC type, and the estimated results showing the SSC-specific associations are listed in Table 2. In general, the association between PM_10_ and death varied in association with the SSC weather conditions prevailing on the day of the mortality event. Specifically, under the same overall PM_10_ level, increased risks of non-accidental death (1.11% (95% CI; 0.50–1.73%)) and cardiovascular death (1.87% (95% CI; 0.68–3.08%)) were observed, for one IQR PM_10_ increase. SSC-specific effects were also detected according to cause of death: in non-accidental deaths, greater and statistically significant associations were observed in DT (1.86% (0.81–2.93%)) and MT (1.86% (1.02–2.70%)), and DT (2.83% (0.74–4.96%)) and MM (3.00% (1.38–4.64%)) showed association with cardiovascular death. Although no overall effect was observed for respiratory death, a modified effect was detected in MT (3.78% (0.89–6.76%)).

### 3.3. Age-Specific PM_10_ Daily Mortality Affected by Weather Classification

Age-specific effects were examined, focusing on spring, and an increased risk for daily mortality, in relation to PM_10_ increase, was observed among those aged >40 years (Table 3). According to the combined SSC types, in terms of humidity, the observed PM_10_ effect in non-accidental deaths was 1.54% (95% CI; 0.09–3.00%) in dry weather type (Dry), and 2.32% (95% CI; 0.74–3.93%) in moist weather type (Moist) among those aged 40–59 years. There was also an increased association between moist weather type and PM_10_ detected across all age groups ≥ 60, as follows: aged 60–74: 1.11% (95% CI −0.12–2.35%); aged 75–84: 1.55% (95% CI 0.26–2.85%); aged 85+: 1.75% (95% CI 0.23–3.28%). In terms of temperature, among non-accidental deaths, the temperature effect was increased in tropical weather type (Tropical) in the 60–74 age group.

An increased association was also typically observed in moist and tropical weather type in cardiovascular deaths among the 75–84 group, with moist weather type showing a risk percentage increase of 2.68% (95% CI 0.42–4.99%), and tropical weather type showing 2.77% (95% CI 0.28–5.33%). In addition, those aged >85 years were more sensitive in transition weather type, which is a highly variable weather condition. For respiratory death, no solid association was detected between PM_10_ level and synoptic weather type.

### 3.4. Weather Classification Influence on the Seasonal Modification of PM_10_ Daily Mortality

Season-specific effects were observed among non-accidental and cardiovascular deaths, according to SSC type (Table 4). A relatively greater, season-specific effect on PM_10_ increase was observed in moist and tropical weather conditions for non-accidental death, and in moist and moderate weather conditions for cardiovascular deaths. For the same level of PM_10_ increase, the effect was detected during summer with tropical weather for non-accidental death (2.59% (95% CI; 1.27–3.93%)) and during winter with moist weather for cardiovascular death (3.69% (95% CI; 1.31–6.13%)). In addition, seasonally differentiated associations were detected according to SSC types, with both non-accidental and cardiovascular deaths showing increased associations during spring (in dry and moderate conditions), in summer (during dry, moist, moderate, and tropical conditions), in fall (during moderate conditions), and in winter (during moist and transition conditions).

## 4. Discussion

### 4.1. Principal Findings

The findings from these analyses suggest that PM_10_ level was influenced by SSC type, in association with an increased risk of mortality. In addition, an increased effect on PM_10_ daily mortality was detected during moderate and tropical weather conditions. In particular, the risk from PM_10_ level increase was dominant in the cardiovascular mortality group, and “moist tropical” weather conditions increased the mortality risk, regardless of the cause of death. In addition, age-specific effects were detected with an increased PM_10_ level in association with moist weather, regardless of age group, among non-accidental deaths, and season-specific effects were also detected in association with SSC type.

### 4.2. Relationships between PM_10_ and Weather Conditions on Mortality

#### 4.2.1. Relationships between Fractions of Particulate Matter and Cause-Specific Mortality

Adverse health effects have recently been reported in terms of particle fraction sizes, as it has become more apparent that the fraction size in the air is an important consideration in PM studies. Particles have been divided into those with an aerodynamic diameter <10 µm (PM_10_), 2.5–10 µm (PM_10–2.5_), or <2.5 µm (PM_2.5_), with PM_10_ being the arithmetical sum of fine particulate matter (PM_2.5_) and coarse particulate matter (PM_10–2.5_). Comparative health effects based on fraction size have been reported to be greater for PM_2.5_ than for PM_10_ for the same unit increase [1,9], and mortality increase associations have also been reported for PM_10–2.5_ [7,8,9,10]. In the current study, we estimated significant associations between PM_10_ and non-accidental mortality and cardiovascular mortality, and although our study was limited in terms of information on the various particle fraction sizes, similar adverse effects were detected, regardless of fraction size, to those studies that differentiated particle fraction sizes [1,7,8,9,10,11]. Controversial findings have been documented, however, with stronger associations for respiratory mortality than for cardiovascular outcomes [11]. Nevertheless, in Yu et al.’s work, the greatest mortality risk was determined to be in hypertensive diseases for both PM_2.5_ and PM_10_, with the detected effect being more robust for PM_2.5_ than for PM_10_ [1]. Regarding the causal pathway from PM exposure to health response, it was suggested that oxidative stress can be stimulated by PM, resulting in damage to both the cardiovascular and respiratory systems [39]. In addition, fine PM can penetrate deep into the lung, damaging the autonomic nervous system and inducing systemic inflammation and oxidative stress, which can then result in increased blood pressure [40]. Accordingly, adverse health effects from elevated PM levels were assigned to hypertensive mortality [1] so that, in relation to our study, similar pathologic effects may be more likely to show among the cardiovascular mortalities, such as stroke [2], myocardial infarction [3], or intracerebral hemorrhage [4], than in respiratory mortality.

#### 4.2.2. Epidemiological Approach for Modified Effect

PM_10_ has been reported to retain its hazardous effect from a few hours to several consecutive weeks. Furthermore, non-accidental mortality increases have been reported from consecutive prolonged events with a high PM_10_ level [41], but effects from weather condition modifications have been reported much less than have PM effects themselves. Modifications [28] or interaction [27,42] between PM and meteorological variables have been investigated, and the data suggested that PM health effects were exacerbated by extreme temperature ranges. In epidemiology, interrelated epidemiologic associations have been considered—in terms of effect modification, interaction, and mediation—which has helped to understand different aspects of diseases or conditions [29,43,44,45]. According to Corraini et al., effect modification aims to separate exposure effects influenced by another variable, while interaction aims to evaluate individual and combined exposure effects [29]. It also has to be considered that a variable may be a step in a chain of events, or pathways, between exposure and outcome, towards being a mediator or an intermediate [29]. In this study, it was hypothesized that PM_10_ health effects were likely to be influenced by synoptic weather conditions, giving rise to different mortality outcomes for the same PM_10_ level; therefore, modified effects for elevated PM_10_ level were estimated, and any distinct health effects based on different SSCs were observed.

#### 4.2.3. Comparisons with Other Studies that Applied the SSC Scheme

The SSC scheme is one approach to be applied; it is a relative and daily weather-type classification scheme rather than an absolute classification system [30] and as such is now widely used in climatological and epidemiological investigations [31,32,33,34,35,36]. Liu et al., after investigating the influence of daily and short term SSC changes on ground-level PM_2.5_ concentrations, suggested that individual meteorological factors affected PM_2.5_ concentrations and reported interactions between atmospheric factors and seasonal and/or geographical factors on PM_2.5_ concentrations [33]. Although the assessment of geographic factors was limited in our study, in fact, PM_10_ concentration was seen to vary according to SSC and season. Among the daily synoptic weather types, DM and DT during overall period; DM (spring), MT (summer), MM and MT (fall), and MM (winter) for season specifically were found to be likely to lead to greater PM_10_ concentration variance. Similarly, greater PM_10_ concentrations were detected under DT conditions during spring and summer, under MT conditions during fall, and under DM conditions during winter. In one Canadian study, varied health effects in relation to SSC types were observed in terms of the association between PM_10_ and asthma hospitalizations, and although the relationship was shown to be not significant statistically, the greatest effect was observed under DT conditions, and statistically significant health effects were noted when MP, DM, and MT conditions prevailed [36].

#### 4.2.4. Varied Study Designs and Adverse Health Effects of Particulate Matters

Two basic study designs have been used to examine PM health effects: a cohort study design, used for long-term effects, [17,19,20], and a time series study design, used to research short-term effects [4,7,9,18,21,22,23,24]. In one, long-term study, marginal associations between PM_10_ and non-accidental mortality were derived from populations using the National Health Insurance Service-National Sample Cohort [19]; the study also observed increased associations among cardiovascular, cerebrovascular, respiratory, and cancer mortalities, although these were statistically insignificant [19].

Our study was based on studying PM_10_ short-term effects, as modified by SSC type, and an increased relative risk of cardiovascular mortality, compared to the risk for general, non-accidental mortality, was observed. Furthermore, PM_2.5_ health effects were higher for cardiovascular disease, cardio-metabolic disease, and ischemic heart disease mortalities, compared to the general health effect of non-accidental causes of death [17]. Regarding respiratory disease, health effects from PM_2.5_ were higher for chronic obstructive pulmonary disease (COPD) mortality, compared to health effects for all non-accidental causes of death [17]. In addition, Wu et al., working in Guangzhou, China, reported that, although ambient PM levels had decreased substantially in Guangzhou recently, cardio-respiratory mortality risks and respiratory mortality risks were still significant and that respiratory mortality risks had even increased in association with PM_2.5_ and PM_10_ [9]. Thus, regardless of study design or PM fraction size, research on adverse health effects and mortality has indicated that it is appropriate to remain wary of PM for ongoing public health protection.

#### 4.2.5. Age-Specified Effects between Particulate Matters and SSC Types

Among the study targets, age-specific effects were reviewed, using four different age subgroups, 40–59, 60–74, 75–84, and 85+. The effect was studied by allocating the estimated effect of elevated PM_10_ levels as a linear explanatory variable in a Poisson regression model for individual causes of death, and the PM_10_ overall effect varied according to age group and cause of death. Among non-accidental mortality, the 40–59 age group was found to have shown more effects from PM_10_ than the other age groups, and within this age group, statistically significant effects on PM_10_ mortality rates were observed in dry, moist, and tropical weather. Among these significant effects, health effect changes related to tropical weather were determined to be the highest. The results indicated a close relationship with temperature, showing strong harmful effects from PM_10_ on non-accidental mortality in connection to temperature range—rather than humidity—among both men (>99th percentile) and women (95–99th percentile) [26].

#### 4.2.6. Weather Characteristics in SSC Types and Relationships between PM_10_ and Cause-Specific Mortality

Looking a little closer at connections between causes of death and weather types and/or seasons, it was apparent that mortality rates increased with elevated temperature and that health effects were greater in extreme temperatures than in moderate temperatures. During summer and winter, when either extremely hot or cold temperatures occurred, tropical weather was strongly associated with non-accidental, cardiovascular, and respiratory mortality. Temperature-related modification of PM_10_ effects has been reported to be higher during high temperatures than during low temperatures [27], and our results were consistent with this, confirming that tropical weather increased the PM_10_ health effect comparatively more than other weather Types during summer. In moist weather, PM_10_ health effects increased during summer, fall, and winter, compared with the overall effects. Moist weather includes moist moderate (MM), moist polar (MP), and moist tropical (MT), and increased adverse effects were predominantly detected among MM and MT periods, for non-accidental and cardiovascular mortality, while MP was shown to be less likely to contribute to increasing adverse effects. Therefore, it was found that modifications caused by the relationship between PM_10_ and temperature were more likely to be seen when there were days with elevated temperature among days already showing extreme hot or cold.

### 4.3. Practical Implications of This Study

In this study, relatively small effects on respiratory mortality were found, whereas in previous work, PM adverse effects on the respiratory system were more clearly detected in medical service utilization, such as emergency department visits [46] or hospital admissions [47]. In the current study, adverse PM effects on respiratory mortality were smaller than the effects on non-accidental mortality or cardiovascular mortality. While similarities were found with previous studies [47,48], relatively greater effects [38] have been reported among cities in China during winter, in a multi-city study that included South Korea, China, and Japan [48]. Cakmak et al., reporting on a long-term PM_2.5_ study in Canada, suggested that long-term, chronic PM_2.5_ exposure could increase lung cancer mortality and that the risk varied spatially by climate zone based on SSC classification [38]. In addition, adverse effects from PM air pollution were likely be found using specific symptoms, such as atopic dermatitis, on a personal exposure basis [37]. Geographically varied, acute effects of PM air pollution, where cause-specific mortality or morbidity are attributed, may require further investigation into just how and at what level PM air pollution health risks are determined and exactly what the influences of different weather conditions are. Applying a weather type classification, such as the SSC scheme, may continue to be convenient in the short term, due to the ease of its application and to its integrated but simplified form.

### 4.4. Study Limitations

Our study has some limitations. Currently, many PM-related studies have moved beyond referring simply to PM_10_ and have considered differential PM fractions or finer particulate matter (PM_2.5_); our study only dealt with PM_10_ due to data acquisition limitations. However, PM health effects have already been reported with little controversy, regardless of fraction size, so it was decided that reviewing health effects without reference to PM fraction size would be unlikely to hinder study outcomes.

In comparing health effects between PM_10_ levels and daily mortality according to synoptic weather types, only limited consideration was given to synoptic weather type prolongation or changes. Prolongation of certain synoptic weather types or changes in synoptic weather types from one to the other in the days prior to a case event may influence the effect of PM, in different seasons, among different population sizes, or may vary with different causes of death.

Finally, the study area was limited to Seoul, and it is necessary to determine, by extending the study area in future work, how our findings can be more broadly applied.

## 5. Conclusions

In our short-term study, it was determined that the PM_10_ influence on mortality—particularly cardiovascular mortality—varied according to SSC type. Use of the SSC system in investigating air pollution and associated short-term health effects assisted our understanding of health risk variation related to weather type. Furthermore, the success in demonstrating the effect of SSC type on modifying PM air pollution suggested that there needs to be a more accentuated focus on synoptic data, as a contributor to acute effect variations in daily mortality.

## Figures and Tables

**Table 1:** Descriptive statistics for daily mortality and environmental variables in Seoul, South Korea, 2000–2014.

Variable		Overall ^a^	Spring	Summer	Fall	Winter
			(Mar.–May) ^a^	(Jun.–Aug.) ^a^	(Sep.–Nov.) ^a^	(Dec.–Feb.) ^a^
Non-accidental mortality		95.4 (12.3)	95.9 (11.3)	89.0 (10.7)	95.5 (11.6)	101.3 (12.5)
Cardiovascular mortality		23.8 (5.7)	24.0 (5.5)	21.6 (5.1)	23.7 (5.7)	25.8 (5.6)
Respiratory mortality		6.7 (2.8)	6.9 (2.7)	6.1 (2.5)	6.3 (2.6)	7.3 (3.1)
Temperature (°C)		12.8 (10.4)	12.1 (6.2)	24.6 (2.5)	14.8 (6.7)	−0.7 (4.6)
Diurnal temperature range (°C)		7.7 (2.7)	8.8 (2.8)	6.8 (2.6)	8.1 (2.6)	7.1 (2.2)
Relative Humidity (%)		61.2 (14.8)	55.7 (14.8)	71.5 (12.2)	62.0 (12.7)	55.6 (13.2)
Air pressure (hPa)		1016.1 (8.2)	1014.7 (6.2)	1007.2 (4.2)	1018.3 (5.6)	1024.3 (5.2)
Rain (mm)		29.5 (145.3)	16.8 (59.1)	52.6 (222.2)	25.1 (95.2)	7.0 (38.3)
PM_10_ (µg/m^3^) ^a^		54.4 (35.0)	67.8 (46.3)	43.3 (25.5)	45.1 (25.9)	61.4 (31.0)
O_3_ (ppb) ^a^		17.1 (14.0)	22.1 (14.7)	22.2 (17.1)	14.3 (10.5)	9.7 (7.3)
NO_2_ (ppb) ^a^		30.1 (18.7)	32.5 (19.2)	24.5 (14.8)	29.8 (18.4)	33.6 (20.5)
Spatial synoptic classification, *N*. of day (%)	Dry moderate (DM)	2037 (37.2)	604 (43.8)	309 (22.4)	737 (54.0)	387 (28.6)
	Dry polar (DP)	1052 (19.2)	194 (14.1)	4 (0.3)	214 (15.7)	640 (47.3)
	Dry tropical (DT)	274 (5.0)	136 (9.9)	101 (7.3)	33 (2.4)	4 (0.3)
	Moist moderate (MM)	868 (15.8)	193 (14.0)	429 (31.1)	152 (11.1)	94 (6.9)
	Moist polar (MP)	217 (4.0)	65 (4.7)	12 (0.9)	43 (3.2)	97 (7.2)
	Moist tropical (MT)	636 (11.6)	61 (4.4)	490 (35.5)	80 (5.9)	5 (0.4)
	Transition (T)	395 (7.2)	127 (9.2)	35 (2.5)	106 (7.8)	127 (9.4)

PM_10_: particulate matters ≤10 μm in aerodynamic diameter; O_3_: Ozone; NO_2_: nitrogen dioxide; ^a^ All exposure variables including pollutant concentrations are presented as the mean (standard deviation) and the overall study period is 5479 days: spring, 1380, summer, 1380, fall, 1365, and winter, 1354 days; ^b^ All presented pollutant concentrations are the means and standard deviations of the daily values.

**Table 2:** Relative risk percentage change between daily mortality and PM_10_ concentration and the modification with synoptic weather type in Seoul, South Korea.

Category	Variables	Relative Risk Percentage Change (95% Confidence Interval) ^a^		
		Non-Accidental Death	Cardiovascular Death	Respiratory Death
Overall effect ^b^	PM_10_	1.11 (0.50, 1.73)	1.87 (0.68, 3.08)	0.83 (−1.27, 2.98)
Modification	Dry moderate (DM)	1.08 (0.41, 1.76)	2.20 (0.88, 3.53)	0.25 (−2.05, 2.60)
	Dry polar (DP)	0.06 (−0.72, 0.85)	0.48 (−1.04, 2.03)	−0.79 (−3.44, 1.93)
	Dry tropical (DT)	1.86 (0.81, 2.93)	2.83 (0.74, 4.96)	−0.24 (−3.83, 3.48)
	Moist moderate (MM)	1.50 (0.67, 2.33)	3.00 (1.38, 4.64)	0.28 (−2.56, 3.19)
	Moist polar (MP)	0.90 (−0.25, 2.06)	1.34 (−0.91, 3.65)	0.17 (−3.70, 4.19)
	Moist tropical (MT)	1.86 (1.02, 2.70)	2.01 (0.37, 3.67)	3.78 (0.89, 6.76)
	Transition (T)	1.00 (0.07, 1.94)	1.91 (0.08, 3.78)	1.89 (−1.29, 5.17)

^a^ Estimated relative risk percent change and 95% confidence interval (CI) of daily mortality by the interquartile increase in PM_10_ of 33.5 µg/m^3^; ^b^ Overall effect of PM_10_ was the estimated effect of the elevated PM_10_ level as a linear term with explanatory variables in the Poisson’s regression model for the cause of individual deaths.

**Table 3:** Age-specified relative risk percentage change between daily mortality and PM_10_ concentration according to SSC types in Seoul, South Korea.

Variables		Age-Specified Relative Risk Percentage Change (95% Confidence Interval) ^a^			
		40–59	60–74	75–84	85+
Non-accidental death	Overall effect ^b^	1.78 (0.38, 3.19)	0.88 (−0.20, 1.97)	1.19 (0.06, 2.33)	1.16 (−0.16, 2.51)
	Dry ^c^	1.54 (0.09, 3.00)	0.74 (−0.37, 1.87)	1.03 (−0.14, 2.20)	0.91 (−0.45, 2.29)
	Moist ^c^	2.32 (0.74, 3.93)	1.11 (−0.12, 2.35)	1.55 (0.26, 2.85)	1.75 (0.23, 3.28)
	Transition ^c^	0.88 (−1.22, 3.04)	1.52 (−0.12, 3.18)	1.06 (−0.64, 2.78)	1.00 (−0.97, 3.02)
	Moderate ^d^	1.28 (−0.23, 2.80)	1.12 (−0.04, 2.29)	1.05 (−0.16, 2.27)	1.70 (0.28, 3.14)
	Polar ^d^	1.12 (−0.62, 2.88)	0.17 (−1.18, 1.53)	0.15 (−1.25, 1.56)	−0.13 (−1.76, 1.52)
	Tropical ^d^	3.07 (1.34, 4.83)	0.98 (−0.37, 2.35)	2.25 (0.84, 3.69)	1.36 (−0.31, 3.06)
	Transition ^d^	0.59 (−1.53, 2.75)	1.51 (−0.14, 3.19)	0.81 (−0.90, 2.54)	1.00 (−0.99, 3.03)
Cardiovascular death	Overall effect ^b^	1.01 (−1.94, 4.05)	1.08 (−0.98, 3.17)	1.75 (−0.24, 3.77)	1.10 (−1.34, 3.60)
	Dry ^c^	1.10 (−1.96, 4.26)	1.22 (−0.90, 3.40)	1.33 (−0.72, 3.41)	0.77 (−1.77, 3.37)
	Moist ^c^	0.92 (−2.41, 4.36)	0.76 (−1.56, 3.14)	2.68 (0.42, 4.99)	1.36 (−1.39, 4.18)
	Transition ^c^	0.11 (−4.40, 4.84)	1.45 (−1.68, 4.67)	0.75 (−2.25, 3.84)	4.00 (0.29, 7.84)
	Moderate ^d^	0.93 (−2.21, 4.17)	1.56 (−0.66, 3.83)	1.77 (−0.37, 3.95)	2.07 (−0.56, 4.77)
	Polar ^d^	1.51 (−2.21, 5.38)	1.15 (−1.46, 3.83)	0.46 (−2.01, 2.99)	−2.02 (−5.05, 1.11)
	Tropical ^d^	0.88 (−2.86, 4.77)	0.26 (−2.29, 2.87)	2.77 (0.28, 5.33)	1.30 (−1.76, 4.47)
	Transition ^d^	0.15 (−4.40, 4.91)	1.63 (−1.53, 4.90)	0.60 (−2.42, 3.72)	3.83 (0.10, 7.69)
Respiratory death	Overall effect ^b^	N/A	1.04 (−3.52, 5.81)	−2.24 (−5.29, 0.92)	−2.24 (−5.29, 0.92)
	Dry ^c^	N/A	1.75 (−2.87, 6.59)	−2.72 (−5.87, 0.52)	−1.88 (−5.46, 1.83)
	Moist ^c^	N/A	−1.20 (−6.37, 4.25)	−1.34 (−4.81, 2.26)	−3.30 (−7.42, 1.00)
	Transition ^c^	N/A	3.78 (−3.02, 11.06)	−1.00 (−5.71, 3.94)	1.16 (−4.08, 6.68)
	Moderate ^d^	N/A	1.51 (−3.32, 6.57)	−2.32 (−5.68, 1.16)	−0.91 (−4.65, 2.97)
	Polar ^d^	N/A	−1.67 (−6.92, 3.89)	−2.81 (−6.59, 1.11)	−4.03 (−8.21, 0.35)
	Tropical ^d^	N/A	4.06 (−1.83, 10.31)	−1.78 (−5.53, 2.12)	−3.18 (−7.85, 1.72)
	Transition ^d^	N/A	3.47 (−3.35, 10.76)	−1.00 (−5.77, 4.02)	1.25 (−4.00, 6.78)

SSC: spatial synoptic classification; N/A: not applicable; ^a^ Estimated relative risk percent change and 95% confidence interval (CI) of daily mortality by the interquartile increase in PM_10_: 33.5 µg/m^3^; ^b^ Overall effect of PM_10_ was the estimated effect of the elevated PM_10_ level as a linear term with explanatory variables in the Poisson’s regression model for the cause of individual deaths; ^c^ Among the seven spatial synoptic classifications, (DM), (DP), and (DT) were combined into “dry,” (MM), (MP), and (MT) into “moist,” and (T) was “transition; ^d^ Among the seven spatial synoptic classifications, (DM) and (MM) were combined into “moderate,” (DP) and (MP) into “polar,” (DT) and (MT) into “tropical,” and (T) was “transition.”

**Table 4:** Relative risk percentage change between daily mortality and PM_10_ concentration according to SSC types in Seoul, South Korea.

Variable	SSC Category	Relative Risk Percentage Change (95% Confidence Interval) ^a^				
		Overall	Spring	Summer	Fall	Winter
Non-accidental death	Dry ^b^	0.94 (0.31, 1.58)	0.97 (0.21, 1.74)	1.77 (0.44, 3.11)	0.80 (−0.46, 2.07)	0.72 (−0.22, 1.66)
	Moist ^b^	1.48 (0.78, 2.18)	0.84 (−0.06, 1.75)	2.18 (0.92, 3.45)	2.30 (0.92, 3.70)	1.63 (0.43, 2.86)
	Transition ^b^	1.12 (0.20, 2.05)	0.74 (−0.50, 2.00)	−1.95 (−5.36, 1.59)	0.88 (−1.07, 2.87)	1.95 (0.51, 3.40)
	Moderate ^c^	1.13 (0.48, 1.79)	1.07 (0.26, 1.88)	1.78 (0.52, 3.06)	1.57 (0.32, 2.84)	1.00 (0.01, 1.99)
	Polar ^c^	0.22 (−0.54, 0.98)	0.33 (−0.73, 1.41)	N/A	0.21 (−1.41, 1.85)	0.21 (−0.84, 1.27)
	Tropical ^c^	1.79 (1.03, 2.56)	1.26 (0.33, 2.20)	2.59 (1.27, 3.93)	2.32 (0.58, 4.09)	N/A
	Transition ^c^	0.98 (0.05, 1.91)	0.81 (−0.44, 2.08)	−1.87 (−5.28, 1.67)	0.97(−0.98, 2.96)	1.75 (0.31, 3.21)
Cardiovascular death	Dry ^b^	1.72 (0.49, 2.97)	2.09 (0.60, 3.60)	2.85 (0.24, 5.52)	2.01 (−0.47, 4.55)	0.73 (−1.11, 2.60)
	Moist ^b^	2.18 (0.82, 3.55)	0.82 (−0.93, 2.60)	3.29 (0.82, 5.82)	2.68 (−0.03, 5.47)	3.69 (1.31, 6.13)
	Transition ^b^	1.90 (0.08, 3.75)	2.05 (−0.40, 4.57)	0.05 (−6.67, 7.24)	−0.37 (−4.21, 3.63)	2.82 (0.01, 5.72)
	Moderate ^c^	2.30 (1.01, 3.60)	2.59 (1.00, 4.21)	3.12 (0.63, 5.67)	2.64 (0.17, 5.17)	1.61 (−0.33, 3.59)
	Polar ^c^	0.63 (−0.85, 2.13)	0.23 (−1.84, 2.34)	N/A	0.90 (−2.26, 4.17)	0.59 (−1.48, 2.70)
	Tropical ^c^	2.15 (0.66, 3.67)	1.33 (−0.50, 3.19)	3.65 (1.04, 6.32)	3.30 (−0.10, 6.82)	N/A
	Transition ^c^	1.84 (0.02, 3.71)	2.20 (−0.28, 4.74)	0.27 (−6.46, 7.50)	−0.38 (−4.23, 3.62)	2.69 (−0.14, 5.60)
Respiratory death	Dry ^b^	0.37 (−1.78, 2.58)	−0.38 (−2.95, 2.26)	−0.30 (−4.85, 4.47)	−0.78 (−5.25, 3.91)	2.33 (−0.81, 5.57)
	Moist ^b^	1.75 (−0.67, 4.22)	−0.08 (−3.16, 3.09)	2.34 (−2.10, 6.98)	3.13 (−1.82, 8.34)	3.59 (−0.51, 7.85)
	Transition ^b^	2.44 (−0.72, 5.71)	2.41 (−1.80, 6.79)	−0.27 (−12.34, 13.46)	−0.85 (−7.50, 6.26)	4.61 (−0.25, 9.71)
	Moderate ^c^	0.40 (−1.85, 2.69)	−0.76 (−3.50, 2.05)	0.83 (−3.57, 5.43)	−0.17 (−4.61, 4.48)	2.86 (−0.43, 6.27)
	Polar ^c^	−0.48 (−3.06, 2.16)	−0.98 (−4.59, 2.76)	−0.79 (−17.97, 19.98)	−0.61 (−6.29, 5.42)	1.05 (−2.47, 4.70)
	Tropical ^c^	2.49 (−0.16, 5.22)	1.29 (−1.86, 4.54)	2.41 (−2.25, 7.29)	5.63 (−0.75, 12.41)	13.57 (−4.33, 34.82)
	Transition ^c^	2.09 (−1.09, 5.37)	2.36 (−1.87, 6.79)	0.00 (−12.12, 13.80)	−0.88 (−7.52, 6.24)	4.14 (−0.72, 9.24)

SSC: spatial synoptic classification; N/A: not applicable. ^a^ Estimated relative risk percent change and 95% confidence interval (CI) of daily mortality by the interquartile increase in PM_10_, 33.5 µg/m^3^. ^b^ Among the seven spatial synoptic classifications, (DM), (DP), and (DT) were combined into “dry,” (MM), (MP), and (MT) into “moist,” and (T) was “transition.’ ^c^ Among the seven spatial synoptic classifications, (DM) and (MM) were combined into “moderate,” (DP) and (MP) into “polar,” (DT) and (MT) into “tropical,” and (T) was transition.”
